# Supplementary material for: Reconciling Mining with the Conservation of Cave Biodiversity: A Quantitative Baseline to Help Establish Conservation Priorities
Source: PLoS One. 2016 Dec 20;11(12):e0168348. doi: 10.1371/journal.pone.0168348 (PMC5173368; doi:10.1371/journal.pone.0168348)
Supplement: S1 Dataset — (ZIP) [file pone.0168348.s002.zip › Taxa/Serra Norte/SN_2013/N4WS/Lista N4WS-23.pdf]

CAVIDADE N4WS-0023

Classe	Ordem	Fam/Outros	Gên/Outros	Espécie	S-U	U-S	Zon.
Arachnida	Araneae	Araneidae		jovem	X		E
Arachnida	Araneae	Theridiosomatidae		jovem	X	X	E
Arachnida	Opiliones	Sclerosomatidae	*Prionostema*	sp.1	X	X	E
Insecta	Diptera	Cecidomyiidae	Cecidomyiinae	sp.		X	E
Insecta	Diptera	Psychodidae	*Sciopemyia*	*sordellii*		X	E
Insecta	Diptera	Tipulidae	Tipulinae	sp.	X	X	E
Insecta	Hymenoptera	Formicidae	*Pachycondyla*	*striata*	X		E
Insecta	Orthoptera	Phalangopsidae	*Paraclodes*	sp.		1	E
Amphibia	Anura	Strabomantidae	*Pristimantis*	cf. *fenestratus*	1		E
Reptilia	Squamata			sp.1	1		E
Reptilia	Squamata			sp.2		1	E
